# Functional Analysis of the Isopentenyl Diphosphate Isomerase of *Salvia miltiorrhiza* via Color Complementation and RNA Interference

**DOI:** 10.3390/molecules201119689

**Published:** 2015-11-10

**Authors:** Xianan Zhang, Hongyu Guan, Zhubo Dai, Juan Guo, Ye Shen, Guanghong Cui, Wei Gao, Luqi Huang

**Affiliations:** 1 School of Traditional Chinese Medicine, Capital Medical University, Beijing 100069, China; zhxn0609@163.com (X.Z.); 13716402100@139.com (H.G.); weigao@ccmu.edu.cn (W.G.); 2 Key Laboratory of Systems Microbial Biotechnology, Tianjin Institute of Industrial Biotechnology, Chinese Academy of Sciences, Tianjin 300308, China; dai_zb@tib.cas.cn; 3 Institute of Chinese Materia Medica, Chinese Academy of Chinese Medical Sciences, Beijing 100700, China; guojuanzy@163.com (J.G.); shenyezy@sina.com (Y.S.); guanghongcui@126.com (G.C.)

**Keywords:** isopentenyl diphosphate isomerase, color complementation, RNA interference, *Salvia miltiorrhiza*, functional identification

## Abstract

Isopentenyl diphosphate isomerase (IPI) catalyzes the isomerization between the common terpene precursor substances isopentenyl diphosphate (IPP) and dimethylallyl diphosphate (DMAPP) during the terpenoid biosynthesis process. In this study, tissue expression analysis revealed that the expression level of the *Salvia miltiorrhiza IPI1* gene (*SmIPI1*) was higher in the leaves than in the roots and stems. Furthermore, color complementation and RNA interference methods were used to verify the function of the *SmIPI1* gene from two aspects. A recombinant *SmIPI1* plasmid was successfully constructed and transferred into engineered *E. coli* for validating the function of *SmIPI1* through the color difference in comparison to the control group; the observed color difference indicated that *SmIPI1* served in promoting the accumulation of lycopene. Transformant hairy root lines with RNA interference of *SmIPI1* were successfully constructed mediated by *Agrobacterium rhizogenes* ACCC 10060. RNA interference hairy roots had a severe phenotype characterized by withering, deformity or even death. The mRNA expression level of *SmIPI1* in the RSi3 root line was only 8.4% of that of the wild type. Furthermore the tanshinone content was too low to be detected in the RNA interference lines. These results suggest that *SmIPI1* plays a critical role in terpenoid metabolic pathways. Addition of an exogenous *SmIPI1* gene promoted metabolic flow toward the biosynthesis of carotenoids in *E. coli*, and *SmIPI1* interference in *S. miltiorrhiza* hairy roots may cause interruption of the 2-*C*-methyl-D-erythritol-4-phosphate metabolic pathway.

## 1. Introduction

*Salvia miltiorrhiza Bunge* is a traditional Chinese medicine that is frequently used for curing cardiovascular diseases, and its preparations are currently in phase II clinical trials [1]. *S. miltiorrhiza* has documented antibacterial, antiphlogosis, thrombosis, myocardial ischemia, hepatic, and antioxidative properties [2], and the tanshinones (such as tanshinone IIA, dihydrotanshinone I, cryptotanshinone, and tanshinone I) are regarded as essential components for these activities. Moreover, *S. miltiorrhiza* has been prominently mentioned in discussions of modern approaches for studying traditional Chinese medicine [3].

**Figure 1:**
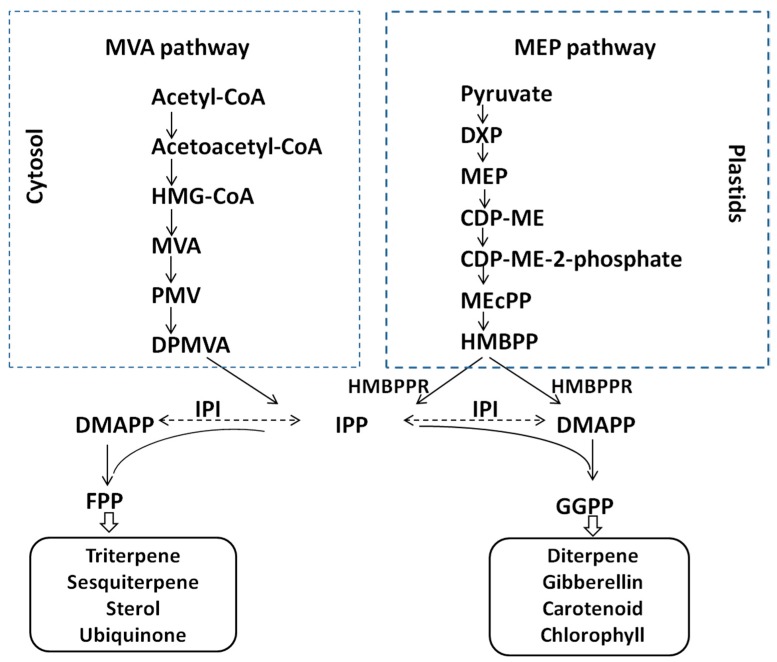
IPP and DMAPP synthesis pathway in plants. DMAPP is converted from IPP by IPI in the MVA pathway, whereas the simultaneous production of IPP and DMAPP from HMBPP occurs in the MEP pathway. HMG-CoA, 3-hydroxy-3-methylglutaryl CoA; MVA, mevalonic acid; IPP, isopentenyl diphosphate; DMAPP, dimethylallyl diphosphate; FPP, farnesyl diphosphate; GAP, glyceraldehyde 3-phosphate; DXP, 1-deoxy-d-xylulose-5-phosphate; MEP, 2-C-methyl-d-erythritol 4-phosphate; HMBPP, 1-hydroxy-2-methyl-2-(*E*)-butenyl 4-diphosphate; HMBPPR, 1-hydroxy-2-methyl-2-(*E*)-butenyl-4-phosphate reductase; GGPP, geranylgeranyl diphosphate.

Tanshinone is a diterpene compound synthesized from two C5 precursors, isopentenyl diphosphate (IPP) or its isomer dimethylallyl diphosphate (DMAPP), which is derived from the mevalonic acid (MVA) pathway in the cytoplasm and the 2-*C*-methyl-d-erythritol-4-phosphate (MEP) pathway in plasmids. In plants, IPP and DMAPP are catalyzed by several terpene synthases and cytochrome P450 enzymes in the downstream pathway to form more than 30,000 kinds of known terpenoids. Isopentenyl diphosphate isomerase (IPI) plays a major role in catalyzing the reversible conversions between the two terpenoid precursors [4] (Figure 1).

IPI is generally categorized into two distinct types: type I and type II. Type I IPI is utilized by most eukaryotes, some bacteria, and certain halophilic archaea, whereas type II IPI has been reported to be produced by bacteria such as *Streptomyces* [5], *Bacillus* [6], *Cyanobacteria* [7] and others. Most plants have two type I IPI isozymes with distinct subcelluar localizations [8]. At present, *IPI* cDNA has been obtained from many species [9,10,11], although their function is not necessarily the same. 

According to previous reports, overexpression of external *IPI* cDNA in prokaryotic cells could lead to a dramatic increase in the content of carotenoids [12,13,14,15]. Silencing of the *IPI* gene in tobacco caused deficiency of photosynthetic pigments, and the reduction of IPI activity affected the synthesis of isoprene substances in tobacco plastids; however, few evident impacts have been observed with respect to the cytoplasmic MVA pathway [16].

In early studies, we selected multiple candidate genes involved in tanshinone biosynthesis in the hairy roots of *S. miltiorrhiza*, as revealed by a cDNA microarray [17] using ~4400 expressed sequence tags, including *SmIPI1*. In order to confirm whether this gene is associated with the synthesis of tanshinones in *S. miltiorrhiza*, we conducted the following experiments: firstly, the *SmIPI1* gene was transferred into lycopene-engineered *Escherichia coli* to determine whether expression of the gene would influence metabolic flow and promote the yield of lycopene. Secondly, RNA interference (RNAi) *SmIPI1* hairy roots lines were established to evaluate the effect of *SmIPI1* on tanshinone accumulation. These studies have the potential to increase our knowledge of the metabolism of terpenes formed through the MEP pathway.

## 2. Results

### 2.1. Sequence Analysis of SmIPI cDNA

**Figure 2:**
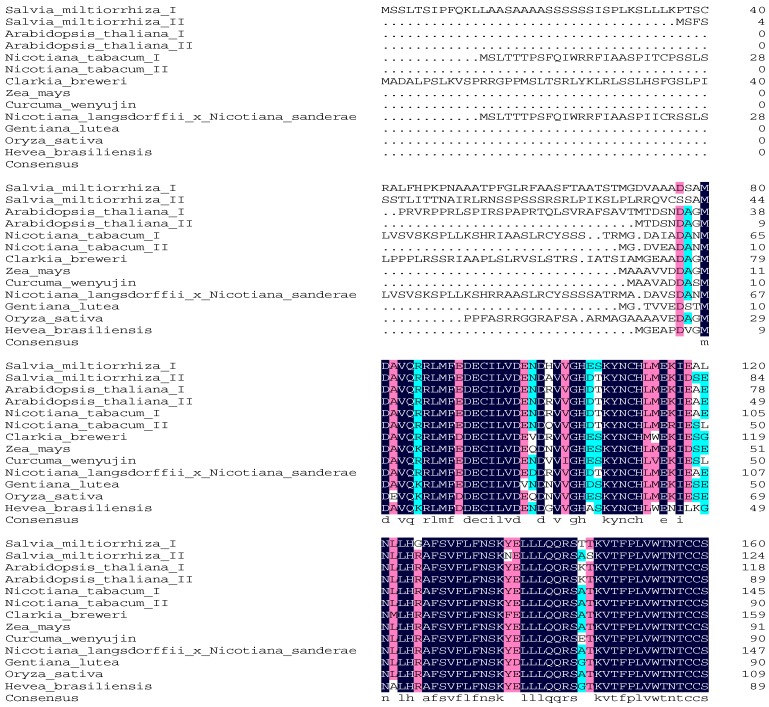

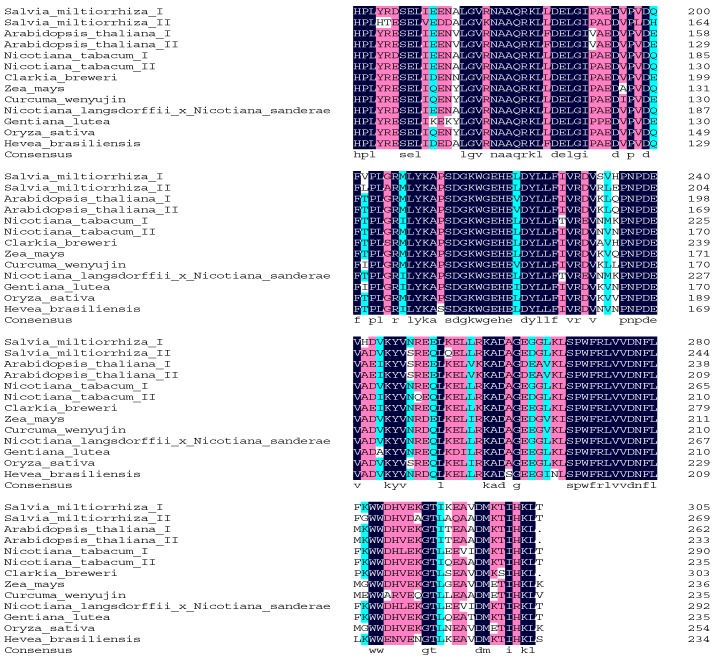
The amino acid sequence alignment of SmIPI1 with IPIs in other plants species. The amino acid accession numbers are as follows: *Arabidopsis thaliana* I AAB67741.1, *Arabidopsis thaliana* II AAL57687.1, *Salvia miltiorrhiza* I ABV08818.1, *Salvia miltiorrhiza* II JN831106.1, *Nicotiana tabacum* I BAB40973.1, *Nicotiana tabacum* II BAB40974.1, *Clarkia breweri* AAB67743.1, *Zea mays* AAQ14869.1, *Curcuma wenyujin* ADE05305.1, *Nicotiana langsdorffii × Nicotiana sanderae* ABB29847.1, *Gentiana lutea* BAE92733.1, *Oryza sativa* AAF29978, *Hevea brasiliensis* AAD41766.1.

**Figure 3:**
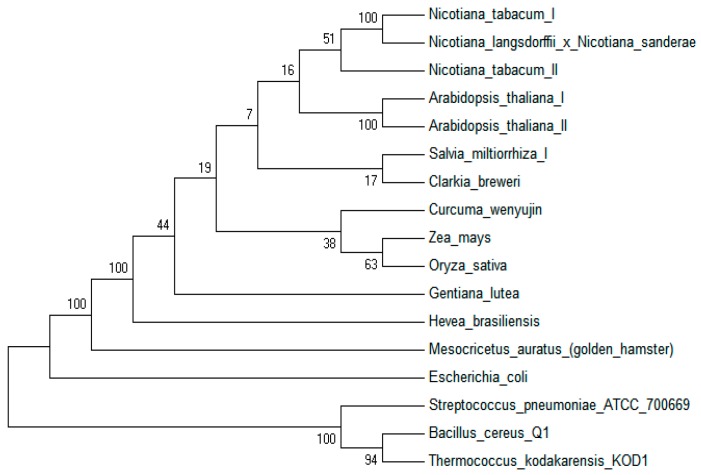
The phylogenetic tree was built based on the partial IPI amino acid sequences of different species using the neighbor-joining method. The amino acid sequence accession numbers are as follows: *Arabidopsis thaliana* I AAB67741.1, *Arabidopsis thaliana* II AAL57687.1, *Salvia miltiorrhiza* I ABV08818. 1, *Nicotiana tabacum* I BAB40973.1, *Nicotiana tabacum* II BAB40974.1, *Clarkia breweri* AAB67743.1, *Zea mays* AAQ14869.1, *Curcuma wenyujin* ADE05305.1, *Nicotiana langsdorffii × Nicotiana sanderae* ABB29847.1, *Gentiana lutea* BAE92733.1, *Oryza sativa* AAF29978, *Hevea brasiliensis* AAD41766.1, *Escherichia coli* AAD26812.1, *Streptococcus pneumoniae* ATCC 700669 B8ZLF5.1, *Bacillus cereus* Q1 B9IVM2.1, *Mesocricetus auratus (golden hamster)* O35586.1, *Thermococcus kodakarensis KOD1* Q76CZ1.1.

The open reading frame of *SmIPI1* cDNA was 918 bp, encoding 305 amino acid (aa) residues, with a relative molecular weight of 34.1 kDa and theoretical isoelectric point of 6.13. The amino acid sequence alignments showed that SmIPI1 has high homology with IPIs of other plant species. SmIPI1 contained a conservative cysteine motif TNTCCSHPL (155–163 aa) and a conservative WGEHELDY motif (217–224 aa) (Figure 2), which belong to the Type I IPIs. The BLAST comparison results indicated that the identity between SmIPI1 and SmIPI2 was 83%, whereas it was 92% and 91% with *Nicotiana tabacum* IPI2 and IPI1, respectively. Relatively higher consistency was also seen with the IPI of *Radix bupleuri* (ACV74320) and *Rhizoma picrorhizae* (AB14800), which was 89% and 90%, respectively. Phylogenetic tree analysis showed that the IPIs of plants were clustered in a bigger branch, SmIPI1 is most closely genetically related with dicotyledon plants (*Arabidopsis thaliana, Nicotiana tabacum etc.*) (Figure 3).

### 2.2. mRNA Expression Analysis of SmIPI1 in Different Organs

**Figure 4:**
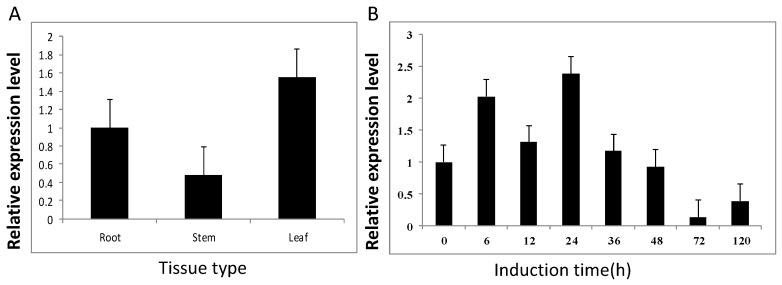
(**A**) Expression pattern of *SmIPI1* in different *S. miltiorrhiza* tissues; (**B**) Expression profile of *SmIPI1* after treatment with 30 mM Ag^+^ over 120 h. The vertical bars show the SD values (*n* = 3).

The *SmIPI1* gene was expressed constitutively in all tissues examined with distinct levels. The highest level of *SmIPI1* mRNA was observed in leaves, followed by roots and stems of *S. miltiorrhiza* (Figure 4A). Several experiments have shown that the addition of various elicitors could increase the yield of plant secondary metabolites to a certain degree, thus, the use of these elicitors are considered to be one of the most effective ways to improve the production of secondary metabolites of medicinal plants. Previous researches have indicated that the Ag^+^ elicitor could promote the accumulation of diterpenoids of *S. miltiorrhiza* [18]. In this study, the relative expression level of *SmIPI1* reached a peak at 24 h after addition of the Ag^+^ elicitor and then declined from 36 to 120 h (Figure 4B).

### 2.3. Color Complementation Analysis of the SmIPI1 Gene

**Figure 5:**
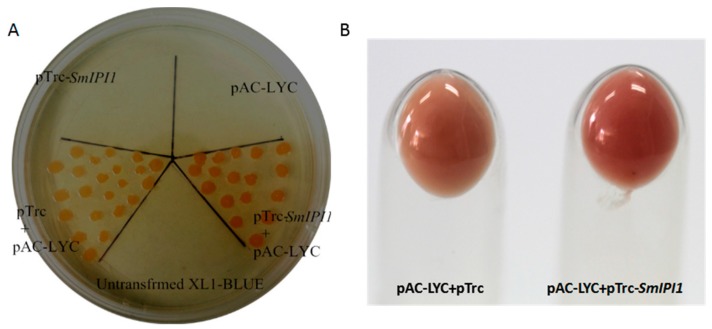
Color complementation and enhanced lycopene accumulation due to expression of *SmIPI1* in engineered *E. coli* for lycopene biosynthesis. (**A**) The plate was divided into five sections, which were respectively inoculated with bacteria containing the corresponding plasmids as indicated on the plate; (**B**) The carroty bacterial clones including pTrc-*SmIPI1* and pAC-LYC were darker because of lycopene accumulation.

The plasmid pAC-LYC, which was transferred into XL1-blue cells, acts to produce β-carotene [19]. In the double-resistant medium, *E. coli* strains harboring pTrc and the pAC-LYC plasmid could grow well, and the bacterial plaques appeared pink. pTrc-*SmIPI1* along with pAC-LYC presented a deeper red color, which likely reflects its ability to accumulate more lycopene by the action of *SmIPI1*. On the other hand, the other three control strains could not grow in this double-resistant medium (Figure 5A). Thus, we can infer that *SmIPI1* can promote the accumulation of lycopene in *E. coli,* and is a key enzyme in the carotenoid biosynthesis pathway.

### 2.4. Confirmation of Transgenic Plants by Fluorescent Microscopy and PCR Analysis

**Figure 6:**
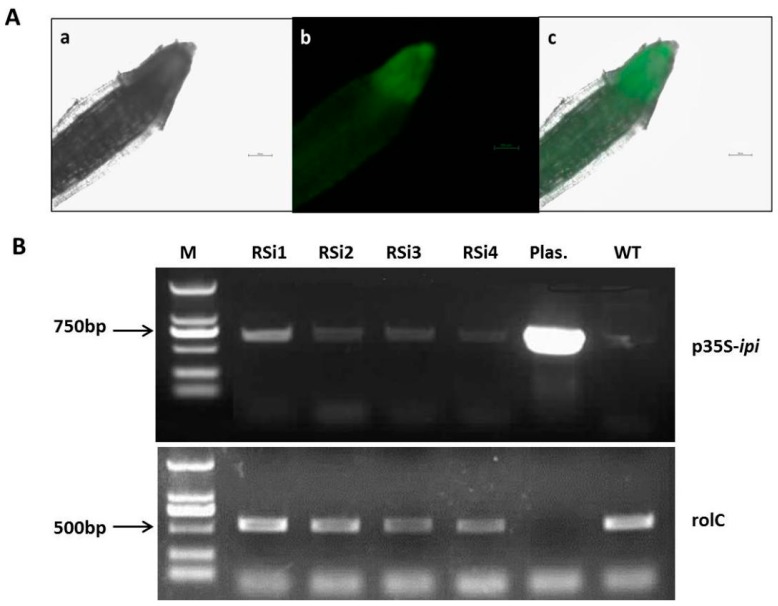
(**Aa**) under white light of fluorescence microscopy; (**Ab**) under blue excitation light of fluorescence microscopy; (**Ac**) superposition of white and blue light of fluorescence microscopy; (**B**) Representative PCR analyses for the presence of p35S-*ipi* and *rolC* genes in transgenic hairy root lines. M: DL-2000 marker (100–2000 bp); RSi: transgenic root lines; WT: the wild type of *S. miltiorrhiza* roots (negative control); Plas: the vector of pK7GWIWG2D-*ipi* (positive control plasmid).

Binary plasmids containing the target gene under the cauliflower mosaic virus 35S promoter were transferred into *S. miltiorrhiza* via *A. rhizogenes* (ACCC 10060). Hairy roots emerged at the edge of the leaf disc after infection with *A. rhizogenes* harboring the binary vector pK7GWIWG2D-*ipi* (RSi). Hairy roots that were brown, thin and shorter were discarded, and only those with good growth capabilities were maintained for further characterization. Hairy roots that were successfully inserted with a target fragment emit a stable and strong green fluorescence signal (520 nm) under the inverted fluorescence microscope with the excitation of blue light (450–490 nm) (Figure 6A). Hairy roots from 1 month old transgenic cultures were used for DNA extraction and PCR-screening. An expected 733 bp fragment of insert sequence (p35S-*ipi*) was amplified in the positive lines in our RNAi lines. WT hairy roots represent negative control and have no insertions and, therefore, no application of the fragment has been observed. The *rolC* gene of 540 bp was amplified (Figure 6B). 

### 2.5. Phenotype, mRNA Expression, and Terpenoid Metabolites Content of RNAi Hairy Roots

**Figure 7:**
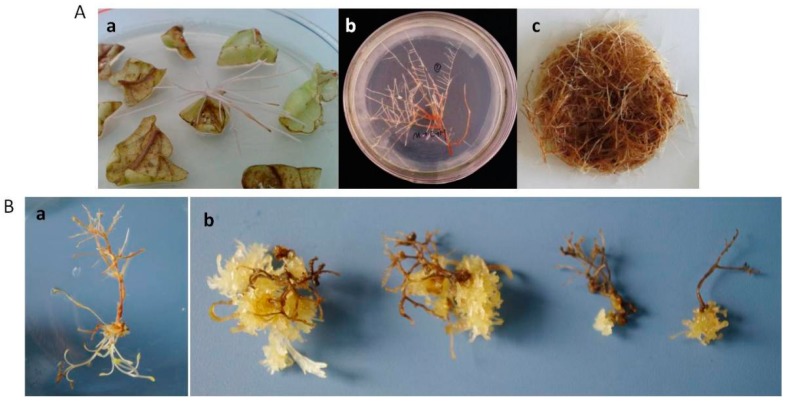
Phenotype comparisons between the RNA interference lines and WT hairy roots. (**Aa**) WT hairy root emerged on the edge of leaves; (**Ab**) single hairy root grew many branches when subcultured on MS solid medium; (**Ac**) WT hairy roots were removed from 6,7-V liquid medium after two month; (**Ba**) single RNAi hairy root was transferred to MS solid medium; (**Bb**) *SmIPI1* interference hairy roots were removed from shake flasks cultures after two months.

*S. miltiorrhiza* hairy roots extended on the edge of leaves (Figure 7Aa), single root was transferred to solid MS medium individually (Figure 7Ab). It was subcultured in shake flasks with 6,7-V liquid medium and then was removed two month later. The WT hairy roots grew denser and brown with lots of branches (Figure 7Ac).

**Figure 8:**
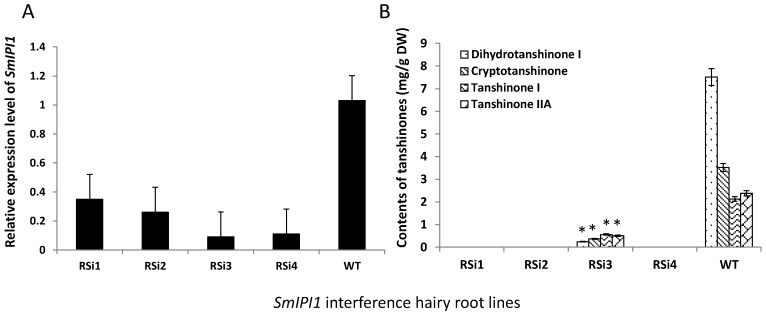
(**A**) The mRNA expression level *SmIPI1* in RNAi hairy root lines and WT; (**B**) Contents of four kinds of tanshinones between RNA interference lines and WT. The values are means ± SD of triplicate analyses. *, significant difference at *p* < 0.05.

On the other hand, the RNAi roots also emerged on the edge of cutting leaves at the beginning, while it grew very slowly when it was separately subcultured on MS solid medium (Figure 7Ba). Cultured for two months in 6,7-V liquid medium, the texture of hairy roots become very brittle, and it grew few branches (Figure 7Bb). Actually a large number of interference lines have been established, but only a few of them survived, most of the hairy roots died in the process of subculture in shake flasks. For the rest, four lines were randomly selected to be monitored. The mRNA expression levels of *SmIPI1* in the four lines were significantly lower than that of the WT. In RNAi line RSi3, it was was only 8.4% of that in the WT roots (Figure 8A), which was the lowest level among the four detected lines. The content of four kinds of terpenoids was detected. The total tanshinone (TT) content in the RNAi line RSi3 was 1.68 mg/g dry weight, which was significantly lower than 15.55 mg/g dry weight in the WT control. The TT contents of the RSi1, RSi2, and RSi4 lines could not be detected (Figure 8B, Table 1). These results suggest that TT accumulation was severely affected due to *SmIPI1* interference. Dihydrotanshinone I, cryptotanshinone, tanshinone I, and tanshinone IIA are the main diterpene tanshinones of *S. miltiorrhiza*, which are generated primarily from the MEP metabolic pathway. It can be preliminary deduced from these results that the IPI1 is essential to the plastid MEP pathway of *S. miltiorrhiza*, and that RNAi of *SmIPI1* in the hairy roots would lead to a sharp reduction of metabolites.

**Table 1:** The tanshinones contents in *S. miltiorrhiza* transgenic hairy roots at the end of the culture time.

Lines	Metabolite contents (mg/g DW)			
	Dihydrotanshinone I	Cryptotanshinone	Tanshinone I	Tanshinone IIA
RSi1	nd	nd	nd	nd
RSi2	nd	nd	nd	nd
RSi3	0.24 ± 0.13 a	0.37 ± 0.28 a	0.56 ± 0.05 a	0.51 ± 0.14 a
RSi4	nd	nd	nd	nd
WT	7.52 ± 0.97c	3.52 ± 0.27 b	2.13 ± 0.12 b	2.38 ± 0.24 b

The values represent the means of three independent experiments (means ± SD). nd, not detected. a–c, different letters indicate significant differences (*p* < 0.05).

## 3. Materials and Methods

### 3.1. Materials

All biological materials used in this work were derived from *S. miltiorrhiza* collected from Shangluo, Shanxi Province, China. Roots, stems, and leaves used for tissue expression pattern analysis were collected from 2-year-old plants in June when the pharmacologically active components were rapidly accumulated [20]. Axenic cultures were prepared after the seeds were surface-sterilized in 0.1% mercuric chloride (Sigma–Aldrich, Shanghai, Trading Co., Ltd., China). Sterile seeds were seeded on Petri dishes containing MS agar medium for germination. Plants were incubated under a 16-h light/8-h dark illumination regime at 25 °C with 60% relative humidity. *S. miltiorrhiza* hairy roots were derived after the seedlings were infected with *Agrobacterium rhizogenes* (ACCC 10060) harboring *Ri* T-DNA. Stock cultures of hairy roots were maintained at 25 °C in the dark on solid hormone-free MS medium with 30 g/L sucrose. For Ag^+^ eliciter treatment, subcultured hairy roots were transferred to 6,7-V liquid medium and cultivated for 7 days. Three mM Ag^+^ elicitor was added to the medium to obtain a final concentration of 30 mM. Hairy roots were harvested at 0, 12, 24, 36, 48, and 72 h, respectively. Each treatment was repeated three times, the samples were dried with filter paper, and then 0.1 g of each sample was stored at −80 °C for RNA extraction. Hairy roots for RNAi experiments were maintained as shake-flask cultures in 250-mL Erlenmeyer flasks, each containing 100 mL medium on an orbital shaker set at 110–120 rpm at 25 °C in the dark. For this study, the synthesis of all primers and DNA sequencing were carried out at the Beijing Sangon Biotechnological (Beijing, China).

### 3.2. DNA and RNA Isolation

Total RNA was extracted from the tissues by Trizol method (Invitrogen, Carlsbad, CA, USA). Genomic DNA of transgenic hairy roots was isolated using the modified cetyltrimethylammonium bromide method [21] for PCR detection.

### 3.3. Bioinformatics Analysis

A BLAST search was conducted to analyze the *SmIPI1* cDNA sequence (GenBank accession number: EF635967) on the National Center for Biotechnology Information website. Sequence homology comparison was performed with ClustalW, and the phylogenetic tree was built using the neighbor-joining method in MEGA5.1.

### 3.4. Quantitative Reverse Transcription-Polymerase Chain Reaction (qRT-PCR) Analysis of SmIPI1 Expression Level

First-strand cDNA was synthesized from 2 μg of total RNA, which was detected by 1% agarose gel electrophoresis according to the manufacturer’s protocol (Takara, Dalian, Biotechnology, Co., Ltd. China). A pair of primers (actinF: 5′-AGGAACCACCGATCCAGACA-3′, actinR: 5′-GGTGCCCTGAGGTCCTGTT-3′) was designed to amplify the housekeeping gene (β-actin) as an internal control, and then the expression level of *SmIPI1* in different tissues was detected with semi-quantitative RT-PCR using another pair of primers (SmI1F: 5′-CGTCCTTGACCAGCATC-3′, SmI1R: 5′-GGCGTTGGGTTTGGGAT-3′). All experiments (including the RNA extraction) were repeated three times.

### 3.5. Reconstruction of the Prokaryotic Expression Vector for Functional Identification of Smipi1 through Color Complementation Analysis

The pTrc-*AtIPI* and pAC-LYC vectors were kindly donated by Francis X. Cunningham Jr. (Department of Cell Biology and Molecular Genetics, University of Maryland, College Park, Baltimore, MD, USA). The pTrc-*AtIPI* vector was the combination of *IPI* gene from *Arabidopsis thaliana* (*AtIPI*), pTrcHisB and pBlueScript SK-. The pTrcHisB vector was digested by restriction enzyme *Kp*nI and *EcoR*I, and then the coding sequence of *AtIPI* inserted, Subsquently, the 6x His tag was removed, as well as the fragment located between *EcoR*V and *EcoR*I. The fragments with an *AtIPI* gene and Trc promoter were finally digested using *EcoR*V and *EcoR*I and inserted into the pBlueScript SK- plasmid. The pTrc-*AtIPI* vector, as an expression vector, retains an *Amp* resistance gene. On the other hand, the pAC-LYC prokaryotic expression vector carries three key enzyme genes, phytoene synthase (crtB), geranylgeranyl pyrophosphate synthase (crtE), and phytoene desaturase (crtI), which encode proteins involved in the β-carotene biosynthetic pathway [22] *E. coli* cells containing pAC-LYC vectors can survive in LB medium with *Chl* and produce small amounts of lycopene, resulting in light pink colonies. For functional analysis of *SmIPI1,* a specific primer pair containing restriction endonuclease sites of *Not*I and *Bgl*II was designed to substitute *AtIPI* in the pTrc-*AtIPI* plasmid as follows: trcIPIF 5′-CATAGATCTATGTCGTCCTTGACCAGCATCCCGT-3′, trcIPIR 5′-ATAGCGGC CTAAGTC-3′. The primers were used for PCR amplification with *SmIPI1* cDNA as a template. The amplified products were purified and introduced into *E. coli* DH5α competent cells, and then selected on Amp (50 mg/L), followed by sequencing analysis. The positive clones with accurate sequencing results were expanded and the plasmid was extracted. pTrc-*AtIPI* was double-digested with *Not*I and *Bgl*II restriction endonucleases and the large fragments ligating with *SmIPI1* were recycled and also doubled-digested with the same endonucleases. The ligation product was named pTrc*-SmIPI1*.The correct pTrc*-SmIPI1* plasmid was extracted after selection on antibiotic plates and then sequenced. The recombinant plasmid pTrc*-SmIPI1* was transformed into XL1-blue competent cells containing the pAC-LYC-engineered bacteria, and streaked onto LB solid medium with *Amp* (100 mg/L) and *Chl* (30 mg/L). Plasmids carrying the double-engineered bacteria that could produce lycopene were obtained. At the same time, the empty vector pTrc and engineered pAC-LYC plasmid were introduced into XL1-blue competent cells to obtain the control strain. Plates were incubated at 37 °C for 24 h and then at 28 °C for 3 days in the dark to allow time for sufficient lycopene accumulation to be able to observe obvious color differences.

### 3.6. Construction of Interfering Plasmids

**Figure 9:**
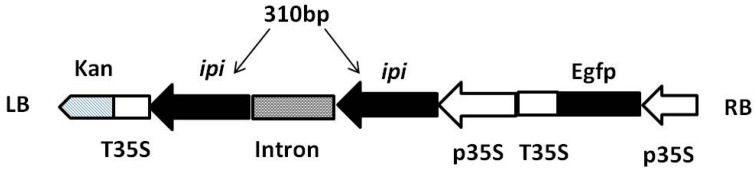
Construction of the pK7GWIWG2D-*ipi* RNA interference vector. p35S, a promoter region from the cauliflower mosaic virus (CaMV) 35S gene; T35S, a terminator fragment from the CaMV 35S gene; Egfp, enhanced green fluorescent protein; *ipi*, 310-bp fragment of *SmIPI1* used for interference plasmid construction; LB, left border; RB, right border.

Based on the protocol of Gateway Clone Technology (Invitrogen), the attB sequence primers RiBF-ipi (5′-GCACAAGTTTGTACAAAAAAGCAGGCTGGACGCCGTTCAGAGGCGCCTC-3′) and RiBR-ipi (5′-GCACCACTTTGTACAAGAAAGCTGGGTTCCTCTGAGCAGCATTCCTC AC-3′) were used to amplify the 310-bp fragment of *SmIPI1* (*ipi*, located from coding sequence positions 240–550 bp) that carries an adjoining attB sequence. Using the right-sequencing attB-*ipi* purified product as a template, the BP clonase II enzyme mix (Invitrogen) was used to construct the entry vector pDONR221-*ipi*. The BP reaction plasmid was used to react with the interfering vector pK7GWIWG2D (Figure 9), adopting the LR clonase II enzyme mix according to the manufacturer’s instructions. Products were transformed into DH5α cells for multiplication with the heat shock method, and then resistance-screened on LB medium containing 50 μg/mL *Spe*. The *Kan*-resistant gene was used as the screening gene and the enhanced green fluorescent protein (Egfp) coding gene was used as the reporter gene owing to its visibility and nondestructive property. Wild-type (WT) hairy roots were regarded as the common control for RNAi lines.

### 3.7. Agrobacterium rhizogenes ACCC 10060 Mediated Gene Transformation into S. miltiorrhiza Hairy Roots

The freeze-thaw method was adopted to transform the plasmid into *A. rhizogenes* ACCC 10060 competent cells. The induction and culturing methods for *S. miltiorrhiza* hairy roots were described previously [23]. Fast-growing antibiotic-resistant hairy roots of 2.0–3.0 cm long were chosen for cutting of a single root and numbered. RNAi hairy roots were screened and cultured on a solid medium of MS containing 400 mg/L *Cef* and 50 mg/L *Kan*.

### 3.8. Green Fluorescent Protein Detection and PCR Verification

The hairy roots growing from the antibiotic plates were placed under an inverted fluorescence microscope to observe green fluorescence excitation. Furthermore, specific primers, 35S-Ripi1 (5′-ATCTAACAGAACTCGCCGTGAA-3′) and 35S-Ripi2 (5′-ACTGAAAGCTCCGTGCAACA-3′), were designed to detect the insertion of the external *ipi* fragment linked to the p35S sequence in the interference lines. The primers RolCF (5′-CTCCTGACATCAAACTCGTC-3′) and RolCR (5′-TGCTTCGAGTTATGGGTACA-3′) were used to detect the *rolC* gene of *A. rhizogenes*, and the positive roots were chosen to be amplified and cultured. 

### 3.9. Measurement of Tanshinones Content

The hairy roots subcultured for 60 days in 6,7-V liquid medium were harvested for tanshinones measurement. The extraction and measuring methods of deterpene components followed those described by Dai *et al.* [23]. The data are expressed as the mean ± SD value of three independent experiments. Comparison of multiple groups was conducted using one-way analysis of variance (ANOVA), and *p* < 0.05 was considered statistically significant.

## 4. Discussion

IPI acts as the general switch of the terpene downstream metabolic pathway, which directly influences the trend of terpenoid metabolic flux [24]. Many studies have demonstrated a significant increase in the content of isoprenes after the transfer of an exogenous *IPI* gene into *E. coli*. Therefore, *IPI* has become an effective target for the metabolic engineering of isoprenes in *E. coli* [25]. However, few studies have examined the *IPI* enzyme in plants. Therefore, we conducted a series of experiments in order to reveal the function of IPI in terpenoid metabolism from several aspects.

In the last decade, Ag^+^ has been reported to be an effective elicitor that could significantly improve the accumulation of tanshinones [26,27]. In our study, the relative expression level of *SmIPI1* showed a parabolic trend of variation over time after induction with Ag^+^, indicating that the tanshinones accumulation of *S. miltiorrhiza* may be closely related to the expression of *SmIPI1*.

The recombinant vector pTrc-*SmIPI1* was verified to facilitate increased lycopene synthesis via the action of a strong promoter, trc, in *E. coli*. Lycopene is a linear carotenoid responsible for the red color in some fruits and vegetables such as tomatoes and watermelon, and contains seven IPP and one DMAPP molecules in its structure. The color of a carotenoid is principally a consequence of the number of conjugated double bonds. As the number of double bonds increases, the carotenoid light absorbance shifts further to the red region of the spectrum [28]. On a dual resistance medium, the bacteria clones containing the double plasmids pAC-LYC and pTrc-*SmIPI1* presented a deeper red color than those containing pAC-LYC and pTrc, which indicated that more lycopene had accumulated. These results confirmed that *SmIPI1* promotes metabolic flux to the biosynthesis pathway of lycopene. According to the recently published genome data of *S. miltiorrhiza* [29], there are two *IPI* cDNAs in *S. miltiorrhiza*. The same method was applied to *SmIPI2* (another homologous *SmIPI* gene) to confirm the action of both genes. Unfortunately, the color of engineered *E. coli* harboring the pTrc-*SmIPI2* plasmid did not show any obvious changes. In *Arabidopsis* and tobacco, it has been demonstrated that the two *IPI* genes (*IPI1* and *IPI2*) were targeted to different organelles and played distinct roles in isoprenoid metabolism [30,31]. The *SmIPI2* gene may also have different functions in *S. miltiorrhiza*, which require further studies for confirmation. 

After genetic transformation of *S. miltiorrhiza* hairy roots, the phenotype of transgenic root lines, changes in mRNA levels of key enzymes, and the metabolic characteristics of RNAi *SmIPI1* lines were analyzed. The gene expression level of *SmIPI1* in the RNAi lines was significantly lower than that of the WT. The fragment at 240–550 bp (corresponding to 80–183 aa) in the *SmIPI1* sequence that we chose for constructing the interference vector is a relatively conserved region with respect to the *IPI* genes of other plants. Therefore, the results obtained in this study likely reflect interference of both *IPI* genes. Interference of *SmIPI1* in the hairy roots resulted in an obvious abnormal phenotype characterized by withering and deformation. It can be inferred that interference of *IPI* gene expression in plants will be a fatal blow to the isoprenoid metabolic pathway, which can seriously affect the growth of plants and interrupt terpenoid metabolism. Maryer *et al.* [16] discovered cell death after knocking out the *IPI* gene (single-copy gene) in *Saccharomyces cerevisiae*. This result illustrated that although the *IPI* protein itself is not required for cell division or spore germination, the cells are unable to survive once the internal pool of isoprenoid metabolites is depleted. Our research is similar to that of the previous study. It contributes novel information to verify that *SmIPI1* plays an important role in the MEP metabolic pathway. Our research will provide a basis for further study on the function of IPI.

## 5. Conclusions

In this study, lycopene engineering bacteria has been used to discover the function of SmIPI1 in *E. coli*. In addition, based on the genetic transformation of *S. miltiorrhiza* hairy roots, phenotype of transgenic hairy roots, changes of mRNA level of *SmIPI1* and metabolic characteristics of *SmIPI1* RNAi hairy roots were analyzed. It is now clearly established that the IPI is crucial in terpenoid synthesis. The study on the IPI function in plants will further figure out the terpenoid metabolic pathway clearly.
